# Safety, short-term patient-reported outcomes, and cosmetic satisfaction of gasless transaxillary endoscopic thyroidectomy: a retrospective analysis of 530 patients

**DOI:** 10.3389/fendo.2026.1911322

**Published:** 2026-08-25

**Authors:** Yifan He, Jie Yuan, Xiyu He, Yawen Bai, Zesheng Zeng, Jianfeng Sheng

**Affiliations:** 1 Department of Thyroid, Head, Neck and Maxillofacial Surgery, The Third Hospital of Mianyang, Sichuan Mental Health Center, Mianyang, Sichuan, China; 2 Affiliated Hospital of North Sichuan Medical College, Nanchong, Sichuan, China

**Keywords:** cosmetic outcomes, endoscopic thyroidectomy, gasless transaxillary approach, patient-reported outcomes, thyroid neoplasms

## Abstract

**Background:**

Gasless transaxillary endoscopic thyroidectomy (GTET) eliminates visible cervical scarring and has been increasingly adopted for managing unilateral thyroid diseases. This study aimed to evaluate its safety, feasibility, perioperative complications, procedure-specific discomfort, and cosmetic satisfaction.

**Methods:**

We retrospectively reviewed 530 consecutive patients with unilateral thyroid disease who underwent GTET between January 2023 and February 2026. To evaluate surgical experience, 406 patients with papillary thyroid carcinoma (PTC) were chronologically divided into early- and late-phase groups (203 cases each). Multivariable linear regression adjusted for baseline confounders. Serum PTH and calcium were measured at 36 hours. Chest wall sensory disturbance and sternocleidomastoid (SCM) discomfort were evaluated at 1 week and 3 months using a modified Verbal Rating Scale (VRS, 0–3), and cosmetic satisfaction via Patient Satisfaction Score (PSS, 0–3).

**Results:**

Among 530 patients (406 PTC, 124 benign), four (0.75%) required conversion to open surgery (two for internal jugular vein bleeding, two for intraoperative recurrent laryngeal nerve transection with immediate neurorrhaphy; none developed permanent paralysis). Postoperative hematoma occurred in six patients (1.13%; one reoperated, five managed conservatively). Two tracheal and one esophageal injury were repaired endoscopically, all recovering within 2 months. Transient vocal cord paralysis occurred in 34 patients (6.42%), permanent in three (0.57%), and transient hypoparathyroidism in 11 (2.08%). Multivariable regression confirmed the late phase independently predicted shorter operative time (β = -14.2 min, P < 0.001) and higher postoperative PTH (β = 4.8 pg/mL, P < 0.001). Chest wall disturbance and SCM discomfort improved significantly from 1 week to 3 months (VRS: 1.41 ± 0.53 vs. 0.36 ± 0.42 and 1.84 ± 0.55 vs. 0.43 ± 0.53, P < 0.001). Over a median follow-up of 25.3 months (IQR: 9.2–34.6), no primary site recurrences occurred. Six patients (1.13%) developed metachronous micro-PTC (0.1–0.4 cm) in the contralateral lobe, successfully treated with completion thyroidectomy.

**Conclusions:**

GTET is a safe and feasible option for carefully selected patients with unilateral thyroid disease, offering favorable cosmetic satisfaction and acceptable morbidity. Procedure-related symptoms are transient. Accumulating surgical experience independently improves operational efficiency and parathyroid preservation.

## 1 Introduction

Thyroid nodules are among the most common endocrine disorders encountered in clinical practice, with approximately 5%-10% of them being malignant (1). In recent decades, the incidence of thyroid cancer has increased steadily worldwide, making it one of the fastest-growing malignancies (2). Papillary thyroid carcinoma (PTC), the most common histological subtype, accounts for more than 90% of all thyroid malignancies and is generally associated with an excellent prognosis (3). Surgical resection remains the primary and most effective treatment for the majority of patients. Complete tumor removal while preserving critical anatomical structures is essential for achieving favorable oncological outcomes and postoperative functional recovery (4).

Conventional open thyroidectomy provides well-established and reliable therapeutic outcomes; however, the permanent cervical scar may negatively affect cosmetic satisfaction, psychological well-being, and health-related quality of life (3). With advances in minimally invasive surgical techniques and increasing patient demand for improved cosmetic outcomes, various remote-access endoscopic thyroidectomy approaches have been developed and widely adopted (5). Among these, gasless transaxillary endoscopic thyroidectomy (GTET) utilizes a concealed incision within the natural axillary crease and creates a working space over the lateral chest wall without continuous CO_2_ insufflation (6, 7). This approach enables complete thyroid resection while avoiding visible cervical scarring and reducing the risk of insufflation-related complications, such as hypercapnia and subcutaneous emphysema (8).

Despite its increasing popularity, concerns persist regarding procedural safety, technical complexity, learning curves, and post-procedural discomfort (9). Large-scale cohort studies rigorously evaluating procedure-specific symptoms and multivariable-adjusted outcomes remain limited. Therefore, we retrospectively reviewed 530 consecutive patients with unilateral thyroid disease who underwent GTET at our institution between January 2023 and February 2026. This study aimed to evaluate perioperative safety, complication profiles, procedure-specific postoperative symptoms, and short-term oncological outcomes within a selected GTET cohort.

## 2 Materials and methods

### 2.1 Study design and patient selection

This single-center retrospective cohort study enrolled 530 consecutive patients with unilateral thyroid disease who underwent GTET at Mianyang Third People’s Hospital between January 2023 and February 2026. All procedures were performed by the same surgical team. The study protocol was approved by the Institutional Ethics Committee of Mianyang Third People’s Hospital (No. 2026S12-1) prior to retrospective data collection, and written informed consent was obtained from all patients. To analyze the effect of sequential surgical experience, the 406 patients with PTC were chronologically divided into an Early-phase group (the first 203 consecutive patients) and a Late-phase group (the subsequent 203 consecutive patients) based on surgical order.

### 2.2 Inclusion and exclusion criteria

Patients were eligible if they met one of the following criteria: (1) unilateral differentiated thyroid carcinoma with a maximum tumor diameter <4 cm, clinically node-negative (cN0) disease, or cN1 disease without matted or fixed lymph nodes; or (2) unilateral benign thyroid nodules requiring surgical treatment with a maximum diameter ≤6 cm. Exclusion criteria included: (1) requirement for total thyroidectomy; (2) radiological evidence of gross extrathyroidal extension or lateral cervical lymph node metastasis (cN1b); and (3) history of previous neck/chest surgery or contraindications to endoscopic procedures. A total of 530 patients met the eligibility criteria and were included in the final analysis, comprising 406 patients with papillary thyroid carcinoma (PTC) and 124 patients with benign thyroid lesions.

### 2.3 Surgical technique

**Figure 1:**
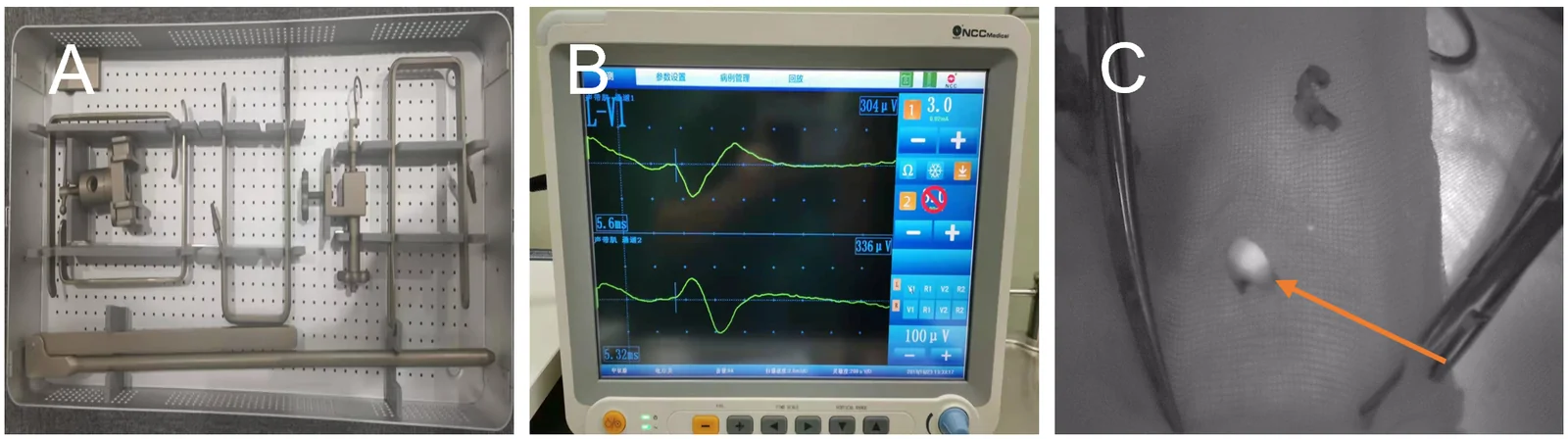
Instruments and monitoring in gasless transaxillary endoscopic thyroidectomy. **(A)** Gasless retractor system for surgical exposure; **(B)** Real-time recurrent laryngeal nerve monitoring; **(C)** Illustrative example of near-infrared parathyroid autogenous fluorescence imaging system selectively applied during surgery (arrow).

**Figure 2:**
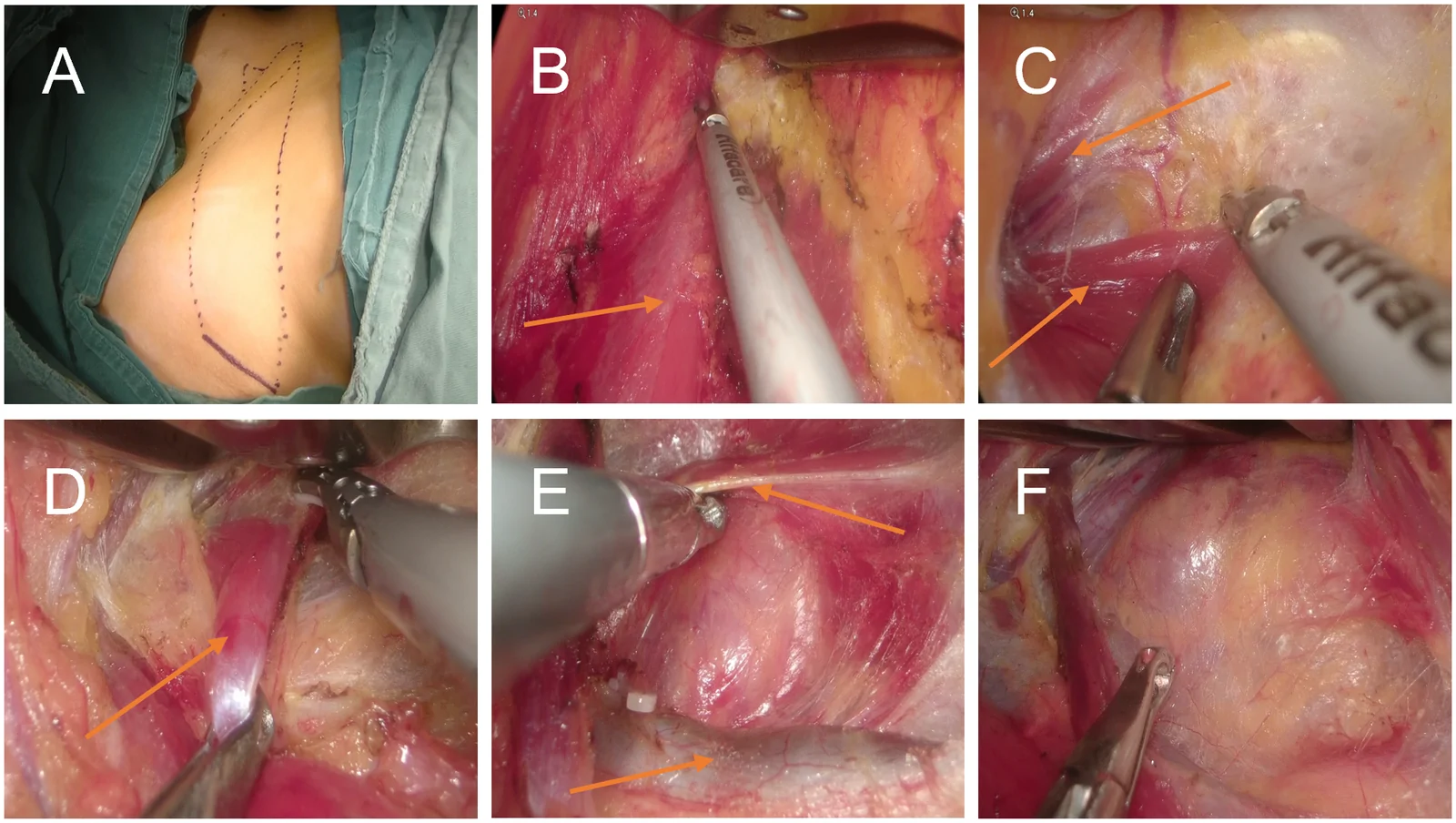
Key steps of gasless transaxillary endoscopic thyroidectomy. **(A)** Preoperative marking of the surgical cavity. **(B)** Dissection of the pectoralis major muscle (arrow). **(C)** Separation of the sternocleidomastoid muscle (upper arrow: sternal head; lower arrow: clavicular head). **(D)** Exposure of the omohyoid muscle (arrow). **(E)** Dissection of the sternothyroid muscle (upper arrow: sternothyroid muscle; lower arrow: internal jugular vein). **(F)** Completed surgical cavity with exposure of the thyroid gland.

The GTET procedure followed standard steps: creation of the working space, central compartment neck dissection (Level VI), and thyroid lobectomy plus isthmusectomy (7, 13). Patients were placed in the supine position with the ipsilateral arm abducted. A 7–8 cm incision was placed along the anterior axillary fold, elevating a subcutaneous flap over the pectoralis major fascia toward the neck. A specialized external retractor system maintained the working space (**Figure 1A**). Dissection proceeded between the sternal and clavicular heads of the sternocleidomastoid muscle (SCM), exposing the omohyoid muscle, internal jugular vein (IJV), and thyroid gland (**Figure 2**). Continuous intraoperative neuromonitoring (IONM) was routinely deployed to identify and preserve the recurrent laryngeal nerve (RLN) (**Figure 1B**).

**Figure 3:**
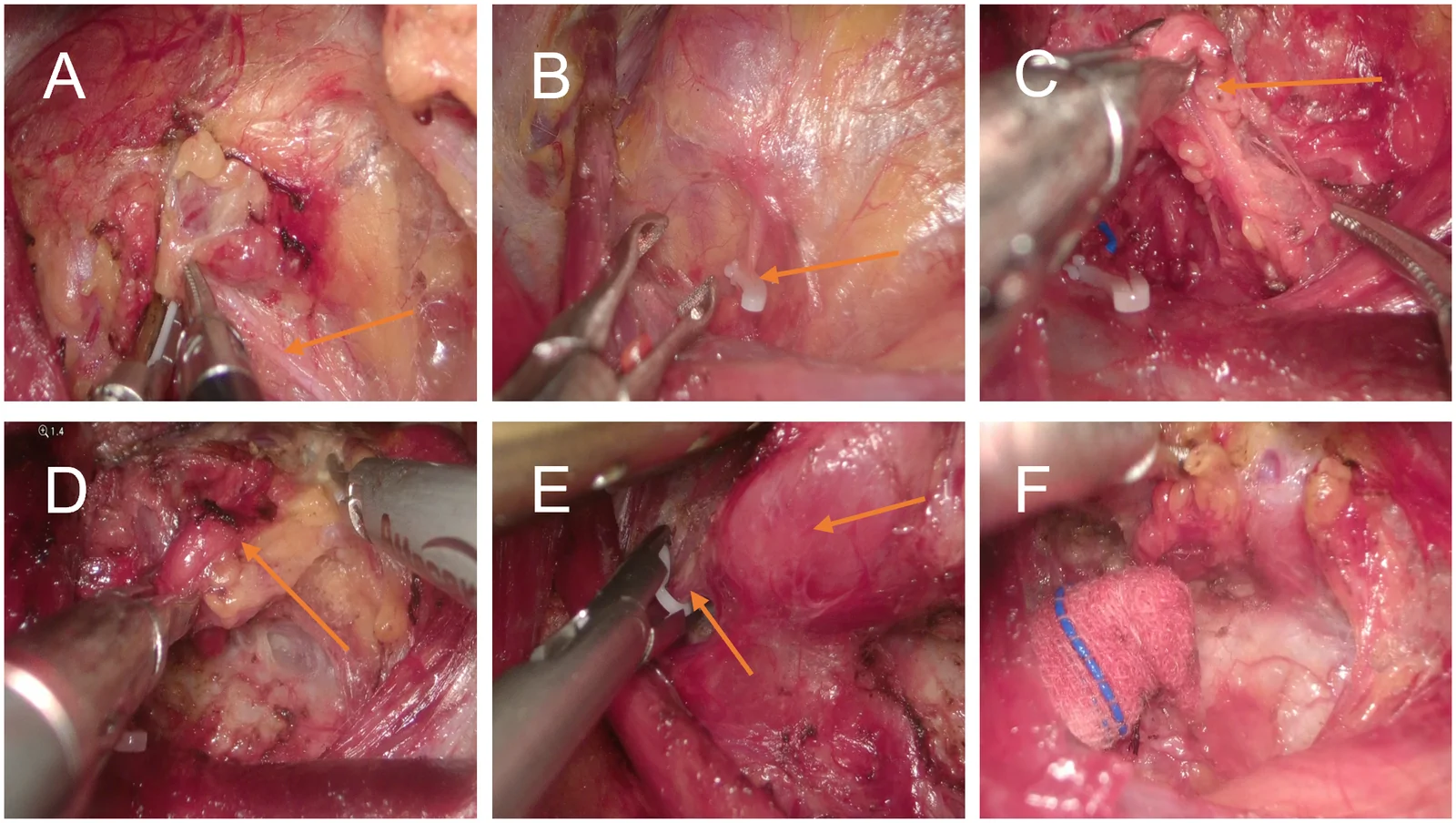
Key intraoperative steps during gasless transaxillary endoscopic thyroidectomy and central lymph node dissection. **(A)** Exposure of the recurrent laryngeal nerve (arrow). **(B)** Ligation of the middle thyroid artery (arrow). **(C)** Dissection of level VIb lymph nodes (arrow). **(D)** Dissection of pretracheal and paratracheal lymph nodes (arrow). **(E)** Clamping of the superior thyroid artery (upper arrow: thyroid gland; lower arrow: superior thyroid artery). **(F)** Separation of the thyroid gland from the trachea.

**Figure 4:**
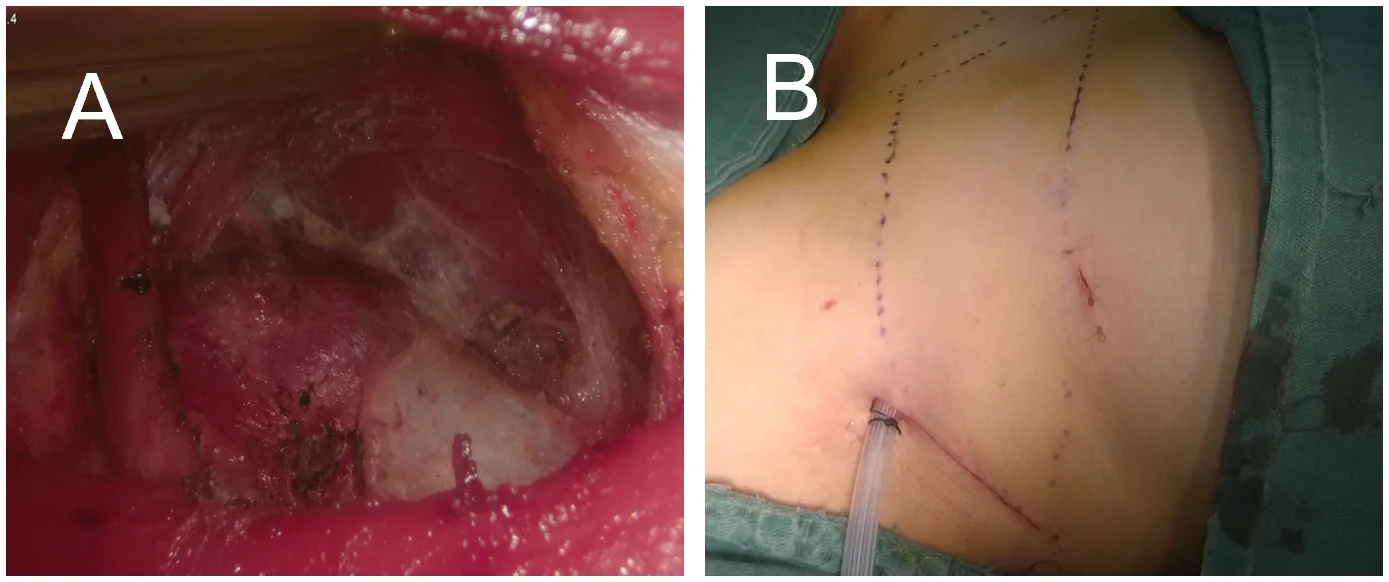
Final intraoperative and postoperative views. **(A)** Surgical cavity after thyroid resection and hemostasis. **(B)** Postoperative appearance showing the axillary incision and drainage tube.

Parathyroid glands with intact vascular supply were preserved *in situ*. Devascularized glands were autotransplanted into the ipsilateral pectoralis major muscle. In cases where parathyroid glands or their microvascular supply could not be clearly identified by direct endoscopic visualization, a near-infrared fluorescence imaging system was selectively deployed to assist in glandular identification and facilitate *in situ* preservation (**Figure 1C**). Central neck dissection encompassing pretracheal, prelaryngeal, and ipsilateral paratracheal compartments was performed under direct RLN visualization (**Figure 3**). Adequate intraoperative hemostasis was performed, and the surgical cavity was closed with sutures (**Figure 4**).

### 2.4 Outcome measures, complications, and follow-up

Primary endpoints included perioperative outcomes and surgery-related complications. Routine indirect or flexible fiberoptic laryngoscopy was performed 48 hours preoperatively for all patients, and postoperatively whenever voice hoarseness or fatigue occurred. Serum parathyroid hormone (PTH) and total serum calcium levels were measured routinely at 36 hours postoperatively. Hypoparathyroidism was defined as PTH < 10 pg/mL. Recovery within 3 months was classified as transient hypoparathyroidism, whereas persistence beyond 3 months was defined as permanent hypoparathyroidism (based on local clinical data showing >90% of parathyroid recoveries occur within 3 months).

Procedure-specific symptoms (anterior chest wall sensory disturbance and SCM discomfort) were evaluated at 1 week and 3 months postoperatively using a modified 0–3 Verbal Rating Scale (VRS: 0 = none, 1 = mild, 2 = moderate, 3 = severe), adapted from published GTET literature. Cosmetic satisfaction was assessed using the 0–3 Patient Satisfaction Score (PSS: 0 = dissatisfied, 1 = neutral, 2 = satisfied, 3 = highly satisfied). All post-procedural assessments were conducted during outpatient visits or telephone follow-ups by trained research assistants who were independent of the operating surgical team.

Postoperative surveillance included serial neck ultrasonography, serum thyroglobulin monitoring, and clinical examination. Contralateral residual lobe events and primary site recurrences were documented.

### 2.5 Statistical analysis

Statistical analyses were performed using SPSS version 28.0. Continuous variables with normal distribution were expressed as mean ± standard deviation (SD) and compared using Student’s t-test. Categorical variables were presented as counts (percentages) and compared using the Chi-square test or Fisher’s exact test. For repeated measurements within the same patient cohort (1 week vs. 3 months), paired t-tests or Wilcoxon signed-rank tests were performed on complete cases (n = 419). To address potential confounding between the early- and late-phase groups, multivariable linear regression models adjusting for age, sex, tumor size, microscopic capsular invasion, tumor laterality, and multifocality were constructed for operative time and postoperative PTH levels. Unadjusted and adjusted regression coefficients (β) with 95% confidence intervals (CIs) were reported. P < 0.05 was considered statistically significant.

## 3 Results

Between January 2023 and February 2026, 530 patients underwent GTET (440 women [83.0%], 90 men [17.0%]; 406 PTC, 124 benign). In the benign cohort, mean tumor size was 1.87 ± 1.37 cm, mean operative time was 136.76 ± 29.05 min, and mean postoperative day 1 (POD1) drainage volume was 47.07 ± 21.18 mL (**Table 1**). In the PTC cohort, mean tumor size was 0.69 ± 0.46 cm, mean operative time was 141.02 ± 25.65 min, and mean POD1 drainage volume was 53.53 ± 25.82 mL (**Table 2**).

**Table 1:** Baseline demographic and perioperative clinical data of 124 patients diagnosed with benign thyroid disease.

Variable	Mean ± SD/n (%)
Age (years)	47.32 ± 10.12
Age group	
≤40 years	31 (25%)
>40 years	93 (75%)
Sex	
Male	15 (12.10%)
Female	109 (87.90%)
Tumor laterality	
Left	56 (45.16%)
Right	68 (54.84%)
Tumor size (cm)	1.87 ± 1.37
Tumor size group	
≤1cm	58 (46.77%)
>1cm	66 (53.23%)
Operative time (min)	136.76 ± 29.05
Estimated blood loss (mL)	20.30 ± 16.80
Drainage volume, POD1 (mL)	47.07 ± 21.18
Length of postoperative hospital stay (days)	6.94 ± 1.02
Drainage duration (days)	3.15 ± 0.55

Continuous variables are shown as mean ± standard deviation; categorical variables are displayed as case number (percentage). POD1, postoperative day 1.

**Table 2:** Baseline demographic and perioperative data of 406 patients with papillary thyroid cancer, divided into early-phase (n=203) and late-phase (n=203) groups.

Variable	Total (n=406)	Early-phase (n=203)	Late-phase (n=203)	*P*	Adjusted *β (95% CI)*
Age (years)	43.17 ± 11.14	42.76 ± 11.44	43.57 ± 10.84	0.464	
Age group				0.484	
≤40 years	177 (43.60%)	85 (41.87%)	92 (45.32%)		
>40 years	229 (56.40%)	118 (58.13%)	111 (54.68%)		
Sex				0.371	
Male	75 (18.47%)	34 (16.75%)	41 (20.20%)		
Female	331 (81.53%)	169 (83.25%)	162 (79.80%)		
Tumor laterality				0.920	
Left	187 (46.06%)	94 (46.31%)	93 (45.81%)		
Right	219 (53.94%)	109 (53.69%)	110 (54.19%)		
Tumor size (cm)	0.69 ± 0.46	0.64 ± 0.45	0.73 ± 0.46	0.047	
Tumor size group				0.013	
≤1cm	344 (84.73%)	181 (89.16%)	163 (80.30%)		
>1cm	62 (15.27%)	22 (10.84%)	40 (19.70%)		
Tumor focality				0.366	
Multifocal disease	73 (17.98%)	33 (16.26%)	40 (19.70%)		
Unifocal disease	333 (82.02%)	170 (83.74%)	163 (80.30%)		
Microscopic capsular invasion				0.022	
Present	183 (45.07%)	80 (39.41%)	103 (50.74%)		
Absent	223 (54.93%)	123 (60.59%)	100 (49.26%)		
Level VIb lymph node metastasis				0.607	
Positive	74 (18.23%)	39 (19.21%)	35 (17.24%)		
Negative	332 (81.77%)	164 (80.79%)	168 (82.76%)		
Operative time (min)	141.02 ± 25.65	149.12 ± 26.85	132.92 ± 21.59	<0.001	-14.2 (-18.6, -9.8) *
Estimated blood loss (mL)	19.60 ± 18.36	20.40 ± 19.47	18.80 ± 16.02	0.366	
Drainage volume, POD1 (mL)	53.53 ± 25.82	53.24 ± 28.42	53.90 ± 22.32	0.795	
Hospital stay (days)	6.90 ± 0.89	7.25 ± 0.80	6.55 ± 0.83	<0.001	-0.68 (-0.84, -0.52) *
Drain duration (days)	3.14 ± 0.58	3.30 ± 0.54	2.99 ± 0.58	<0.001	-0.31 (-0.42, -0.20) *
Harvested lymph nodes (n)	5.31 ± 3.41	5.75 ± 3.58	4.86 ± 3.17	0.008	-0.89 (-1.55, -0.23) *
Pre-op PTH (pg/mL)	59.48 ± 23.58	60.49 ± 24.16	58.45 ± 22.94	0.383	
Post-op PTH (pg/mL)	34.34 ± 15.98	31.55 ± 14.79	37.12 ± 16.67	<0.001	4.8 (2.1, 7.5)*
Post-op calcium (mmol/L)	2.23 ± 0.12	2.24 ± 0.13	2.21 ± 0.11	0.012	

Multivariable regression coefficients (β) adjusting for age, sex, tumor size, capsular invasion, laterality, and multifocality are included for key continuous outcomes. Pre-op, preoperative; Post-op, postoperative; PTH, parathyroid hormone. POD1, postoperative day 1.

Regarding surgical safety across all 530 patients, four (0.75%) required conversion to open surgery: two due to uncontrollable IJV hemorrhage and two for immediate microsurgical neurorrhaphy following intraoperative RLN transection. Neither of the two repaired RLN cases developed permanent vocal cord paralysis during follow-up. Intraoperative endoscopic repair was successfully achieved for two tracheal injuries and one esophageal injury; all three patients recovered fully within 2 months without fistulae. Postoperative hematoma occurred in six patients (1.13%); one required emergency reoperation for active bleeding, while five were managed conservatively via compression and percutaneous aspiration. Transient vocal cord paralysis occurred in 34 patients (6.42%), permanent in 3 (0.57%), and transient hypoparathyroidism in 11 (2.08%). No surgical site infections were recorded (**Table 3**).

**Table 3:** GTET surgery-related complications, procedure-specific symptoms, and cosmetic outcomes split by surgical diagnosis and follow-up intervals.

Variable	Mean ± SD/n (%)	Benign cohort (n=124)	PTC cohort (n=406)	P-value/change
Conversion to open surgery, n (%)	4 (0.75%)	0 (0.00%)	4 (0.99%)	0.582
Thermal skin injury, n (%)	2 (0.38%)	1 (0.81%)	1 (0.25%)	0.326
Internal jugular vein injury, n (%)	7 (1.32%)	1 (0.81%)	6 (1.48%)	0.684
Recurrent laryngeal nerve transection, n (%)	2 (0.38%)	0 (0.00%)	2 (0.49%)	1.000
Transient hypoparathyroidism, n (%)	11 (2.08%)	0 (0.00%)	11 (2.71%)	0.082
Postoperative hematoma, n (%)	6 (1.13%)	1 (0.81%)	5 (1.23%)	1.000
Tracheal injury (endoscopic repair), n (%)	2 (0.38%)	0 (0.00%)	2 (0.49%)	1.000
Esophageal injury (endoscopic repair), n (%)	1 (0.19%)	0 (0.00%)	1 (0.25%)	1.000
Vocal cord paralysis, n (%)				
Transient	34 (6.42%)	5 (4.03%)	29 (7.14%)	0.228
Permanent	3 (0.57%)	0 (0.00%)	3 (0.74%)	0.569
Anterior chest wall sensory disturbance (VRS score)				
one week postoperatively (n=503)	1.41 ± 0.53	1.38 ± 0.51	1.42 ± 0.54	Paired P < 0.001*
three months postoperatively (n=419)	0.36 ± 0.42	0.34 ± 0.40	0.37 ± 0.43	(Vs 1 week)
Sternocleidomastoid muscle discomfort (all patients)				
one week postoperatively (n=503)	1.84 ± 0.55	1.81 ± 0.52	1.85 ± 0.56	Paired P < 0.001*
three months postoperatively (n=419)	0.43 ± 0.53	0.40 ± 0.50	0.44 ± 0.54	(Vs 1 week)
Sternocleidomastoid muscle discomfort (VRS score)				
one week postoperatively	1.84 ± 0.55	1.81 ± 0.52	1.85 ± 0.56	Paired P < 0.001*
three months postoperatively (n=419)	0.43 ± 0.53	0.40 ± 0.50	0.44 ± 0.54	(Vs 1 week)
Cosmetic satisfaction (PSS score)				
one week postoperatively (n=503)	2.88 ± 0.39	2.89 ± 0.38	2.88 ± 0.39	Paired P = 0.528
three months postoperatively (n=419)	2.90 ± 0.35	2.91 ± 0.34	2.90 ± 0.35	(Vs 1 week)

Assessments conducted by independent trained assessors using modified VRS (0–3) and PSS (0–3) scales. Paired t-test (or Wilcoxon signed-rank test) comparing scores at 3 months versus 1 week postoperatively across the total followed-up cohort. Other P-values represent intergroup comparisons between the Benign and PTC cohorts.

Comparing the early-phase (n = 203) and late-phase (n = 203) PTC groups, the late-phase group had significantly larger tumors (0.73 ± 0.46 vs. 0.64 ± 0.45 cm, P = 0.047) and a higher rate of microscopic capsular invasion (50.74% vs. 39.41%, P = 0.022). Despite greater tumor complexity, the late-phase group demonstrated significantly shorter operative times (132.92 ± 21.59 vs. 149.12 ± 26.85 min, P < 0.001), shorter drainage durations (2.99 ± 0.58 vs. 3.30 ± 0.54 days, P < 0.001), and higher postoperative PTH levels (37.12 ± 16.67 vs. 31.55 ± 14.79 pg/mL, P < 0.001) (**Table 2**).

Multivariable linear regression confirmed that after adjusting for age, sex, tumor size, capsular invasion, tumor laterality, and multifocality, the surgical phase remained an independent factor associated with shorter operative time (β = -14.2 min, 95% CI: -18.6 to -9.8, P < 0.001) and higher postoperative PTH levels (β = 4.8 pg/mL, 95% CI: 2.1 to 7.5, P < 0.001).

Follow-up was available for 503 patients at 1 week and 419 patients at 3 months. The overall median follow-up duration was 25.3 months (IQR: 9.2–34.6 months), with 357, 283, and 113 patients followed for at least 1, 2, and 3 years, respectively. Mean VRS scores for anterior chest wall sensory disturbance decreased significantly from 1.41 ± 0.53 at 1 week to 0.36 ± 0.42 at 3 months (paired P < 0.001). SCM discomfort scores decreased from 1.84 ± 0.55 to 0.43 ± 0.53 (paired P < 0.001). Late-phase patients reported lower SCM discomfort than early-phase patients at 1 week (1.78 ± 0.55 vs. 1.92 ± 0.54, P = 0.010). Cosmetic satisfaction was high (PSS score: 2.88 ± 0.39 at 1 week, 2.90 ± 0.35 at 3 months).

No local or regional recurrence occurred at the primary operative site during follow-up. Six patients (1.13%) were diagnosed with metachronous micro-PTC (0.1–0.4 cm) in the contralateral residual thyroid lobe between 12 and 19 months postoperatively. All six underwent successful completion thyroidectomy (2 N1a, 4 N0) without distant metastasis.

## 4 Discussion

Remote-access endoscopic thyroid surgery has gained wide acceptance for avoiding visible cervical scars while delivering therapeutic outcomes comparable to open thyroidectomy (10, 11). In this retrospective study of 530 consecutive patients undergoing gasless transaxillary endoscopic thyroidectomy (GTET), we demonstrated that GTET can be safely performed in carefully selected patients with unilateral thyroid disease, yielding acceptable operative outcomes, low rates of major complications, excellent cosmetic satisfaction, and favorable short-term oncological results.

One of the major barriers to the widespread adoption of GTET is its relatively steep learning curve (12). Compared with conventional open thyroidectomy, GTET requires the creation of a large working space, maintenance of flap retraction, and dissection through an unfamiliar anatomical perspective (13). In our chronological subgroup analysis, later surgical experience was independently associated with reduced operative time and better parathyroid preservation despite managing larger tumors with higher capsular invasion rates. Multivariable regression confirmed that surgical phase independently contributed to a 14.2-minute reduction in operative time and a 4.8 pg/mL increase in post-op PTH. These findings indicate that procedural familiarity enhances surgical efficiency and parathyroid preservation. For novice endoscopic surgeons, initial case selection should prioritize small (≤1 cm), unifocal, cN0 lesions in the left thyroid lobe to avoid the added anatomical complexity of dissecting the right Level VIb compartment beneath and posterior to the RLN.

Establishment of the working space represents one of the most technically demanding aspects of GTET (14). Injury to the IJV is a well-recognized complication during transaxillary thyroid surgery and remains a major cause of conversion to open surgery, particularly during the early learning phase (15). In the current series, seven patients experienced IJV injury; however, only two required conversion to open surgery because endoscopic hemostasis could not be achieved. Notably, all conversions related to IJV injury occurred in the early-phase group. This finding suggests that improvements in anatomical orientation and endoscopic surgical skills significantly reduce the impact of vascular injury on operative outcomes. Therefore, careful patient selection, meticulous preoperative evaluation, and structured surgical training are crucial during the initial learning period to ensure procedural safety (16).

Preservation of RLN and parathyroid gland function remains a fundamental indicator of thyroid surgery quality (17). IONM enables the identification of nerves through electrodes. By measuring nerve responses, the electrical field is converted into acoustic signals, facilitating nerve localization and preventing damage during surgery (18). In our cohort, rates of transient vocal cord paralysis and transient hypoparathyroidism were within acceptable ranges and comparable to those reported in previous studies (7, 13). Most affected patients recovered during follow-up, suggesting that transient traction injury, local edema, or thermal effects rather than permanent structural damage were responsible for the majority of postoperative dysfunction. Of particular interest, two patients experienced intraoperative RLN transection and underwent immediate microsurgical repair after conversion to open surgery; neither subsequently developed permanent vocal cord paralysis. These findings highlight the importance of routine IONM and meticulous surgical technique in preserving postoperative function.

Oncological safety remains the most critical concern regarding GTET for thyroid cancer (19). Adequate central neck dissection is essential for optimal locoregional control, particularly in patients with papillary thyroid carcinoma (20). Unlike the left side, where the esophagus provides a predictable anatomical bed, the right side features a larger posterior space behind the trachea (21). In the gasless transaxillary approach, the right RLN acts as a physical barrier. Accessing the right level VIb compartment requires working beneath and posterior to the nerve in a narrow, one-dimensional tunnel, which maximizes the risk of traction and thermal injury (22). In our institution, routine use of IONM allows precise localization of the RLN and facilitates comprehensive dissection of the paratracheal compartment while minimizing neural injury. During the study period, metastases within the VIb compartment were identified in 74 patients, indicating that GTET can provide sufficient exposure for standardized central neck dissection. Furthermore, no definite locoregional recurrence was observed during the available follow-up period. Given the predominance of low-risk PTC and the relatively limited follow-up duration, these oncological findings should be interpreted cautiously. Longer-term follow-up remains necessary to confirm the oncological equivalence of GTET.

Unlike conventional open thyroidectomy, GTET requires creation of an extensive anterior chest flap and prolonged retraction of the SCM, which may result in procedure-specific postoperative symptoms (23). Procedure-specific symptoms such as chest wall numbness and SCM discomfort were common early postoperatively but improved substantially by 3 months. These transient symptoms stem from subcutaneous flap elevation and retractor pressure rather than permanent nerve injury. Moreover, patients treated during the later phase group reported significantly less SCM discomfort than those treated earlier. This observation indicates that postoperative functional symptoms are influenced not only by the surgical approach itself but also by surgeon experience, flap design, and retraction duration (3).

Cosmetic benefit remains one of the primary motivations for choosing GTET (9). More than 97% of patients in our study reported satisfaction or high satisfaction with their postoperative appearance. By concealing the incision within the natural axillary crease, GTET completely avoids visible cervical scarring, an advantage that may be particularly important for young and cosmetically conscious patients (24). Previous studies have demonstrated that cervical scars may adversely affect self-image, social interactions, and long-term quality of life (25). Therefore, the value of GTET extends beyond cosmetic improvement alone and may positively influence psychological well-being and patient-reported quality of life (7).

Several limitations must be noted. First, this was a single-center retrospective study lacking a contemporaneous open thyroidectomy control group. Second, procedure-specific symptoms were evaluated using simple unvalidated surgical rating scales. Third, long-term follow-up (>3 years) was available for only a subset of patients (n=113), precluding definitive long-term oncological equivalence conclusions. Future prospective comparative studies with extended follow-up are warranted.

## 5 Conclusion

GTET is a safe, feasible, and cosmetically favorable option for carefully selected patients with unilateral thyroid disease. Procedure-related chest wall and SCM discomfort are transient and improve markedly over time. Accumulating surgical experience independently contributes to improved operative efficiency and parathyroid preservation. Future multicenter prospective studies with longer follow-up are needed to confirm the long-term safety and oncological efficacy of GTET.

## Data Availability

The raw data supporting the conclusions of this article will be made available by the authors, without undue reservation.
